# Antigen-Presenting Cell-Like Neutrophils Foster T Cell Response in Hyperlipidemic Patients and Atherosclerotic Mice

**DOI:** 10.3389/fimmu.2022.851713

**Published:** 2022-02-17

**Authors:** Tingrui Zhao, Qingsong Jiang, Wenming Li, Yin Wang, Yao Zou, Xinyu Chai, Zhiyi Yuan, Limei Ma, Ruihong Yu, Tao Deng, Chao Yu, Tingting Wang

**Affiliations:** ^1^ College of Pharmacy, Chongqing Medical University, Chongqing, China; ^2^ Chongqing Key Laboratory for Pharmaceutical Metabolism Research, Chongqing, China; ^3^ Chongqing Pharmacodynamic Evaluation Engineering Technology Research Center, Chongqing, China; ^4^ Chongqing Key Laboratory of Biochemistry and Molecular Pharmacology, Chongqing, China; ^5^ Department of Clinical Laboratory, University-Town Hospital of Chongqing Medical University, Chongqing, China; ^6^ Research Center of Pharmaceutical Preparations and Nanomedicine, College of Pharmacy, Chongqing Medical University, Chongqing, China

**Keywords:** neutrophils, APC-like phenotype, CD3^+^ T cells, interferon-γ, atherosclerosis

## Abstract

Neutrophils constitute abundant cellular components in atherosclerotic plaques. Most of the current studies are focused on the roles of granular proteins released by neutrophils in atherosclerosis. Here, we revealed a unique subset of neutrophils which exhibit the characteristics of antigen-presenting cell (APC) (which were called APC-like neutrophils afterwards) in atherosclerosis. The roles of APC-like neutrophils and relevant mechanisms were investigated in hyperlipidemic patients and atherosclerotic mice. Higher percentages of neutrophils and APC-like neutrophils were found in peripheral blood of hyperlipidemic patients than that of healthy donors. Meanwhile, we also identified higher infiltration of neutrophils and APC-like neutrophils in atherosclerotic mice. Ox-LDL induced Phorbol-12-myristate-13-acetate (PMA)-activated neutrophils to acquire the APC-like phenotype. Importantly, upon over-expression of APC-like markers, neutrophils acquired APC functions to promote the proliferation and interferon-γ production of CD3^+^ T cells *via* HLA-DR/CD80/CD86. In accordance with what found *in vitro*, positive correlation between neutrophils and CD3^+^ T cells was observed in hyperlipidemic patients. In conclusion, our work identifies a proinflammatory neutrophil subset in both hyperlipidemic patients and atherosclerotic mice. This unique phenotype of neutrophils could activate the adaptive immune response to promote atherosclerosis progression. Thus, this neutrophil subset may be a new target for immunotherapy of atherosclerosis.

## Introduction

Atherosclerosis is a chronic inflammatory disease that occurs in the walls of blood vessels, and is the pathological basis for cardiovascular diseases (1). Its morbidity and mortality rates are higher than those of any other diseases in the world (2). Hyperlipidemia, especially hypercholesterolemia, leads to accumulation of plasma low-density lipoprotein (LDL) in the artery wall; LDL and its components elicit vascular inflammation that drives the build-up of lipid-laden atherosclerotic plaques (3). Hyperlipidemia is one of the risk factors for atherosclerosis (4) and represents the initiation stage of atherosclerosis (5). The accumulation of lipids in the arterial wall, as well as the infiltration of a large number of inflammatory cells such as macrophages, neutrophils and T cells, are the main characteristics of atherosclerosis (6, 7). The atherosclerotic lesion contains lipid oxides such as oxidized LDL (ox-LDL) (8) and a variety of inflammatory cytokines. The innate immune response and adaptive immune response both play important roles in the genesis of atherosclerosis (9). The innate immune system is triggered by the activation of vascular endothelial cells (10, 11) and monocytes/macrophages (12),while the adaptive immune system is initiated by antigen-presenting cells presenting multiple antigens to effector T cells (13).

Neutrophils are the most abundant leukocytes in peripheral blood and one of the first innate immune cells to arrive at the site of inflammation (14). Neutrophils contribute to the occurrence and progression of atherosclerosis by releasing granular proteins such as matrix metalloproteinase (15, 16), myeloperoxidase (17, 18), elastase (19) and forming neutrophil extracellular traps (NETs) (20, 21). There are a growing body of evidences suggesting that neutrophils have a highly variable transcriptome profile depending on their tissue location and microenvironment (22). As a result, under the condition of different stimulating factors, neutrophils may exhibit different phenotypes and exert different functions. For example, in the tumor microenvironment, tumor-associated neutrophils are proposed to be polarized into an anti-tumor or pro-tumor phenotype (23, 24). In allergic diseases, IL-33 can stimulate neutrophils to produce Th1 cytokines such as IL-5, IL-9, IL-13, and so on, thus promotes disease progression (25). Neutrophils could possess different functions and phenotypes depending on the disease model (26–29).

Antigen presenting cells (APCs) are required for the priming of adaptive immune system (30). APCs uptake and present antigens to T cells, triggering the adaptive immune response (31). The major histocompatibility complex (MHC), and the costimulatory molecules CD80 and CD86, are the key molecules responsible for T cell activation (31, 32). Strikingly, the presence of APC-like neutrophils has been discovered in infectious diseases (33), allergic diseases (28), and tumors (34). For instance, in patients with rheumatoid arthritis, neutrophils in the synovial fluid express large amounts of class II MHC molecules and then stimulate T cell proliferation (35). APC-like neutrophils gain the ability to activate T cells, resulting in the production of inflammatory cytokines, and ultimately, promote or dampen disease progression. So, whether APC-like neutrophils exist in atherosclerotic plaques and the potential roles of this unique neutrophil subset in atherosclerosis are of great importance for further understanding of this inflammatory disease.

Herein, we show that APC-like neutrophil subset exists in peripheral blood of hyperlipidemic patients and atherosclerotic plaques of LDLr^-/-^ mice. Upon exposing to ox-LDL, PMA-activated neutrophils upregulate the expression of HLA-DR, CD80 and CD86, exhibiting an APC-like phenotype. In turn, APC-like neutrophils enhance the proliferation and interferon-γ (IFN-γ) production of CD3^+^ T cells *via* HLA-DR/CD80/CD86. Clinical data show a positive correlation between APC-like neutrophils and CD3^+^T cells, which implies APC-like neutrophils may promote atherosclerosis progression through activating adaptive immune system.

## Materials and Methods

### Human Samples

Peripheral blood of 90 hyperlipidemic patients were collected from University-Town Hospital of Chongqing Medical University. Hyperlipidemic patients with high blood pressure or diabetes mellitus were excluded. Peripheral blood of 90 healthy donors were used as control. Informed consent was signed by each subject. The study was approved by the Ethics Committee of Chongqing Medical University.

### Mice

Six-to-eight-week-old male LDLr^-/-^ mice and C57BL/6 mice (wild-type mice, WT mice) were purchased from Beijing huafukang Biotechnology Co. All mice were bred in specific pathogen-free conditions. All animal experiments were undertaken with review and approval from the Animal Ethical and Experimental Committee of Chongqing Medical University.

### Atherosclerosis Mouse Model

LDLr**^-/-^** mice were fed with high-fat-diet (HFD) containing 0.15% cholesterol (medicine Ltd, China) for 12 weeks to generate lipid-induced atherosclerosis (LDLr^-/-^ HFD). WT and LDLr^-/-^ mice fed with normal diet were used as control (WT ND and LDLr^-/-^ ND). Each group contained eight mice. After feeding for 12 weeks, LDLr^-/-^ mice and WT mice were anesthetized and sacrificed. Blood in heart and aorta were flushed with sterile phosphate-buffered saline (PBS)-sodium heparin solution *via* cardiac puncture. Hearts were excised and fixed with 4% paraformaldehyde. Aortas were digested for 1 h at 37°C using an enzyme mixture containing 450 U/ml collagenase Is (Sigma-Aldrich, USA), 125 U/ml collagenase XI (Sigma-Aldrich, USA), 60 U/ml DNase I (Sigma-Aldrich, USA), and 60 U/ml hyaluronidase (Sigma-Aldrich, USA) as previously reported (36). Mouse peripheral blood was collected in the Eppendorf tubes containing Heparin sodium. Digested aorta, bone marrow from femur and tibia and spleen were prepared into a single cell suspension by grinding for further use.

### Mouse Blood Lipids Analysis

Plasma samples were collected from LDLr^-/-^ mice and WT mice for lipids measurement. Total cholesterol (TC), total triglyceride (TG), low-density lipoprotein-cholesterol (LDL-C) and high-density lipoprotein-cholesterol (HDL-C) in mouse plasma were assayed with corresponding assay kit (Mindray, China) by using biochemical analyzer (Mindray, China).

### Assessment of Atherosclerotic Lesion

Frozen sections of aortic sinuses were stained with Oil-red O (Solarbio, China) to determine the lipids deposition. Total lesion areas defined as intimal atherosclerotic areas and lesion lipid deposition areas were evaluated by Image J. Paraffin-embedded hearts were cut into 6-8-μm thick slides for Hematoxylin-Eosin staining and Masson’s staining to visualize the necrotic core areas and the collagen contents. Necrotic core areas and collagen contents of atherosclerotic lesions were measured by ImageJ.

### Isolation of Neutrophils and CD3^+^ T Cells

Peripheral blood was obtained from healthy adult volunteers. Human neutrophils were isolated by density gradient centrifugation using Ficoll (Solarbio, China) according to manufacturer’s instructions. The purity of neutrophils was up to 98% (**Supplementary Figure S1A**). Peripheral blood mononuclear cells (PBMC) from healthy donors were isolated by density gradient centrifugation using Ficoll (Solarbio, China). CD3^+^ T cell from PBMC were sorted by Easysep™ human T cell isolated kit (Stemcell, Canada). The viability of sorted CD3^+^ T cells was higher than 90% and their purity was up to 97% (**Supplementary Figure S1B**).

### Neutrophil Stimulation

Freshly isolated neutrophils were treated with ox-LDL, PMA, ox-LDL plus PMA, respectively. As for PMA and ox-LDL plus PMA groups, neutrophils were pretreated with 1 nmol/L PMA (Solarbio, China) for 30 min and the cells were washed with RPMI1640 medium. Then the cells were incubated with 40 μg/ml ox-LDL (Yiyuanbiotech, China) for 12h, 24h, 36h, 48h, separately, and harvested for flow cytometric analysis.

### *In Vitro* Neutrophil-T Cell Co-Culture System

Purified CD3^+^ T cells (3×10^6^ cells/ml) were labelled with carboxyfluorescein succinimidyl ester (CFSE) and co-culture with neutrophils pre-stimulated with ox-LDL, PMA, or ox-LDL plus PMA at a 1:1 ratio in 200 μL RPMI-1640 medium containing rhIL-2 (20IU/mL) (Peprotech), anti-CD3 (2 μg/mL) (Biolegend), and anti-CD28 (1 μg/mL) (Biolegend) antibodies, with or without human CD80 neutralizing antibody (20 μg/mL) (Biolegend), human CD86 neutralizing antibody (20 μg/mL) (Biolegend) and human HLA-DR neutralizing antibody (20 μg/mL) (Biolegend). After 5-day incubation, the cells were collected for intracellular cytokine staining.

### Flow Cytometry

Flow cytometric analysis was performed according to standard protocols. Cell surface markers were stained with fluorescence labeled antibodies for 30 min at 4°C. Anti-human CD45, CD66b, CD80, CD86, HLA-DR, CD11c antibodies were applied to analyze the phenotype of neutrophils in hyperlipidemic patients and healthy donors. Anti-mouse CD45, Ly6G, CD80, CD86, MHC-II antibodies were applied for detecting neutrophil phenotype in atherosclerotic mice. For intracellular staining, the cells were stimulated for 6 hours with Cell Activation Cocktail with Brefeldin A (Biolegend, USA) (5). Intracellular cytokine staining was performed after the cells were fixed and permeabilized with fixation/permeabilization buffer (eBioscience, USA) for 20 min (37). Anti-human CD45, CD3, IFN-γ antibodies and anti-mouse CD45, CD3, IFN-γ antibodies were used for intracellular cytokine staining. All flowcytometric antibodies except anti-IFN-γ antibody (Invitrogen, USA)were purchased from Biolegend (USA).

### Statistical Analysis

Each experiment was performed at least three times. Data were expressed as mean ± SEM. Differences between hyperlipidemic patients and healthy donors were analyzed by the unpaired, Student’s test. Correlations between parameters were assessed using the Pearson correlation analysis and linear regression analysis as appropriate. The animal data and *in vitro* data were analyzed by non-parametric analysis. GraphPad Prism 7.0 was used for all statistical analysis. All data were analyzed using two-tailed tests, and p<0.05 was considered statistically significant.

## Results

### Neutrophils Increase in Peripheral Blood of Hyperlipidemic Patients and Express Characteristic Markers of APCs

**Figure 1:**
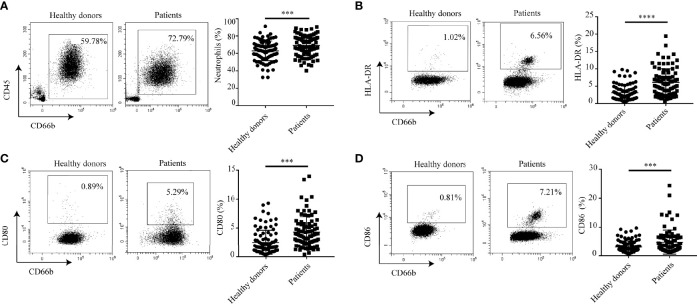
Neutrophils express characteristic surface molecules of APCs in hyperlipidemic patients. **(A)** The percentage of neutrophils in peripheral blood of patients with hyperlipidemia and healthy donors. **(B–D)** Flow cytometry analysis of the percentages of HLA-DR^+^, CD80^+^, CD86^+^ neutrophils between patients with hyperlipidemia and healthy donors. ***p < 0.001, ****p < 0.0001.

To identify neutrophil subsets in hyperlipidemic patients, we first used flow cytometry to analyze the percentage of CD45^+^CD66b^+^ neutrophils within the total CD45^+^ leukocytes in different samples from 90 hyperlipidemic patients. 90 healthy donors were used as control. It showed that patients with hyperlipidemia had a greater proportion of neutrophils in peripheral blood than healthy donors (**Figure 1A**). Since neutrophils in patients with cancer or infectious diseases have the ability to heterogeneously express some co-stimulatory molecules, we postulated that there might be a subset of neutrophils with characteristics of APCs in atherosclerosis. So, the expression of APC-like markers on neutrophils were examined by flow cytometry. As expected, increased percentages of peripheral neutrophils in hyperlipidemic patients expressed APC signature markers including HLA-DR (**Figure 1B**), CD80 (**Figure 1C**) and CD86 (**Figure 1D**), but few neutrophils expressed dendritic cell (DC) marker CD11c (**Supplementary Figure S2**). Further analysis of the co-expression of HLA-DR, CD80 and CD86 on neutrophils showed that the percentage of HLA-DR^+^ CD80^+^ CD86^+^ neutrophils in peripheral blood of hyperlipidemic patients is significantly higher than that of healthy donors (**Supplementary Figure S3**). These results imply that a subset of APC-like neutrophils exists in hyperlipidemic patients. As most of the atherosclerosis cases developed from hyperlipidemia, we speculate that APC-like neutrophils may play pivotal roles in atherosclerosis.

### APC-Like Neutrophils Are Enriched in Murine Atherosclerosis

To assess the distribution of APC-like neutrophils in atherosclerosis, we established a murine model of atherosclerosis by feeding LDLr^-/-^ mice on a high-fat diet (HFD) for 12 weeks (LDLr^-/-^ HFD cohort). LDLr^-/-^ mice and WT mice fed with normal diet (ND) were set as control groups (LDLr^-/-^ ND cohort; WT ND cohort). Oil Red O staining of the whole aorta from aortic arch to abdominal aorta showed that LDLr^-/-^ mice with HFD had increased atherosclerotic areas than control groups (**Supplementary Figure S4A**). Oil Red O staining of aortic root also showed the same trend (**Supplementary Figure S4B**). Masson trichrome staining and HE staining showed increased collagen contents and enlarged necrotic core areas appeared in aortic root of LDLr^-/-^ mice with HFD (**Supplementary Figures S4C, D**). Blood lipid levels including TC, TG, LDL-C and HDL-C of LDLr^-/-^ mice with HFD were significantly higher than control groups (**Supplementary Figure S4E**). Besides, the weight of LDLr^-/-^ mice fed with HFD was much higher than control (**Supplementary Figure S4F**). These results indicate that the mouse model of atherosclerosis is established successfully.

**Figure 2:**
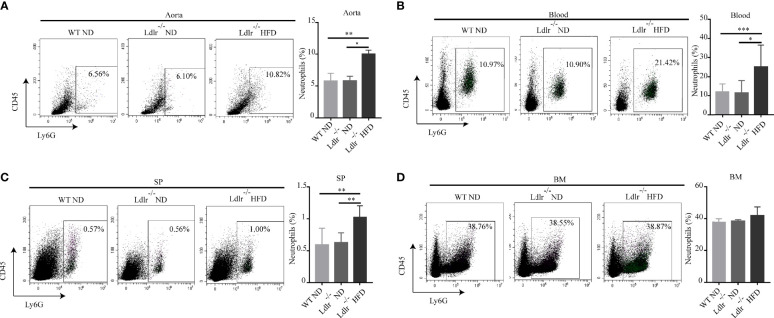
Percentage of neutrophils was higher in LDLr^-/-^HFD mice than control groups. Distribution of neutrophils in **(A)** aorta, **(B)** blood, **(C)** spleen (SP), **(D)** bone marrow (BM) of atherosclerotic mice and control groups were detected by flow cytometry. n=8. *p < 0.05, **p < 0.01, ***p < 0.001.

**Figure 3:**
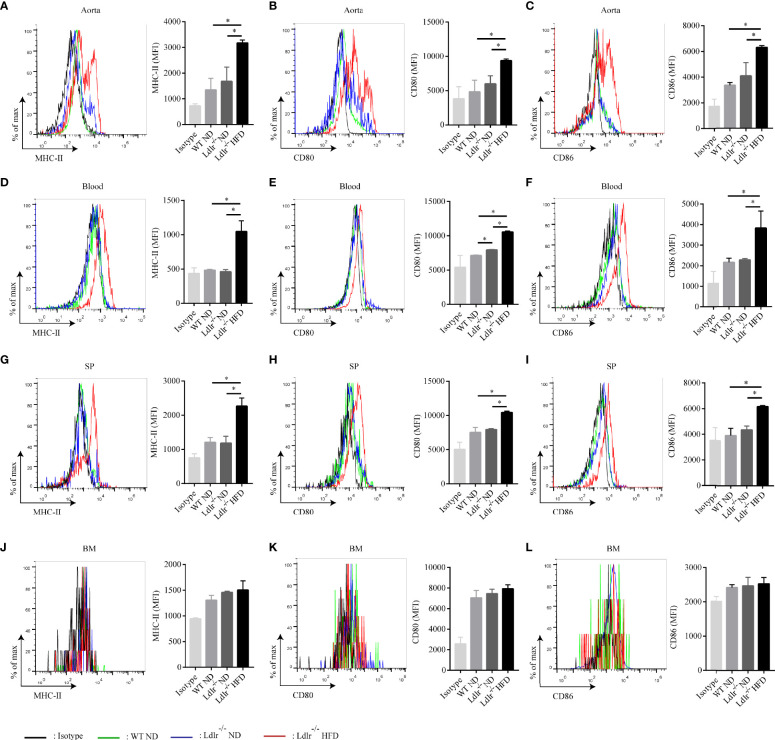
Neutrophils with characteristics of APC are present in atherosclerotic mice. Expression of MHC-II, CD80, CD86 on neutrophils in the **(A–C)** aorta, **(D–F)** blood, **(G–I)** spleen (SP), and **(J–L)** bone marrow (BM) of WT ND (green line), LDLr^-/-^ ND (blue line) and LDLr^-/-^ HFD mice (red line) (n=8). Black line: isotype control. MFI, mean fluorescence intensity. *p < 0.05.

Then, the distribution of neutrophils in aorta, blood, spleen and bone marrow of atherosclerotic mice were analyzed by flow cytometry. As shown in **Figure 2A**, neutrophils were enriched in aorta of LDLr^-/-^ mice feeding with HFD. Similarly, atherosclerotic mice showed a higher neutrophil percentage in peripheral blood (**Figure 2B**) and spleen (**Figure 2C**) than control groups. However, no such differences were observed in the bone marrow (**Figure 2D**). Next, the subsets of neutrophils in different tissues were detected. In accordance with what found in hyperlipidemic patients, neutrophils in aorta of LDLr^-/-^ mice with HFD showed higher expression of APC-like markers including MHC-II molecules (**Figure 3A**), CD80 (**Figure 3B**) and CD86 (**Figure 3C**). The same trends were also observed in neutrophils from peripheral blood (**Figures 3D–F**) and spleen (**Figures 3G–I**) of atherosclerotic mice, while neutrophils in the bone marrow expressed none such markers (**Figures 3J–L**). Taken together, these results clarify the existence of APC-like neutrophils in atherosclerotic mice. The different distribution of APC-like neutrophils between peripheral tissues and bone marrow suggests that the APC-like neutrophils may be regulated under hyperlipidemic conditions other than generated in the bone marrow.

### Ox-LDL Induces PMA-Activated Neutrophils Differentiating Into APC-Like Phenotype

**Figure 4:**
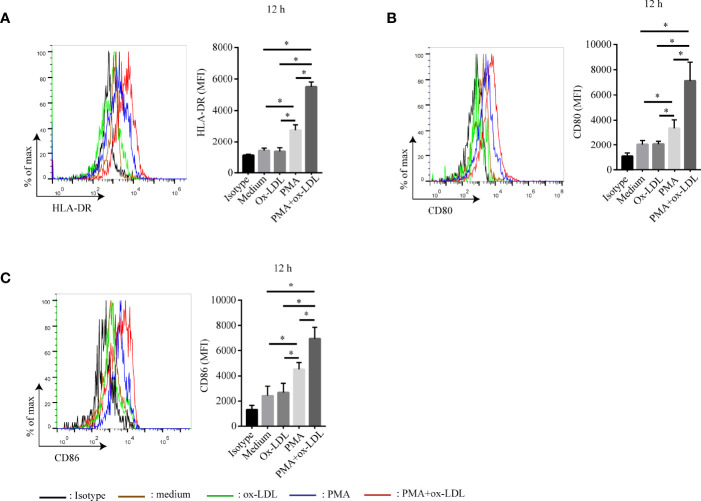
Ox-LDL induces PMA-activated neutrophil to express HLA-DR, CD80 and CD86. Isolated neutrophils were treated under different conditions: no treatment (brown line), 40 μg/ml ox-LDL alone (green line), 1 nmol/L PMA (blue line), 40 μg/ml ox-LDL plus 1 nmol/L PMA (red line). After 12h, expression of **(A)** HLA-DR, **(B)** CD80, **(C)** CD86 on neutrophils were analyzed by flow cytometry. Black line: isotype control. *p < 0.05.

Since ox-LDL is one of the most important lipids in atherosclerosis, we wonder whether it could modulate the differentiation of APC-like neutrophils. To verify this hypothesis, neutrophils were isolated from peripheral blood of healthy donors and stimulated with ox-LDL, PMA, ox-LDL plus PMA for 12 hours, respectively. Surprisingly, neutrophils treated with ox-LDL alone showed little expression of HLA-DR, CD80, and CD86 (**Figures 4A–C**). However, these markers were dramatically upregulated on neutrophils treated with ox-LDL plus PMA (**Figures 4A–C**). The same results were observed when neutrophils were stimulated for 24h, 36h, 48h, respectively (**Supplementary Figure S5**). These findings demonstrate that when neutrophils are activated by PMA, they can differentiate into APC-like phenotype by ox-LDL, which implies other factors in atherosclerosis may work together with ox-LDL to regulate the differentiation of APC-like neutrophils.

### APC-Like Neutrophils Possess the Ability to Activate T Cell Response

**Figure 5:**
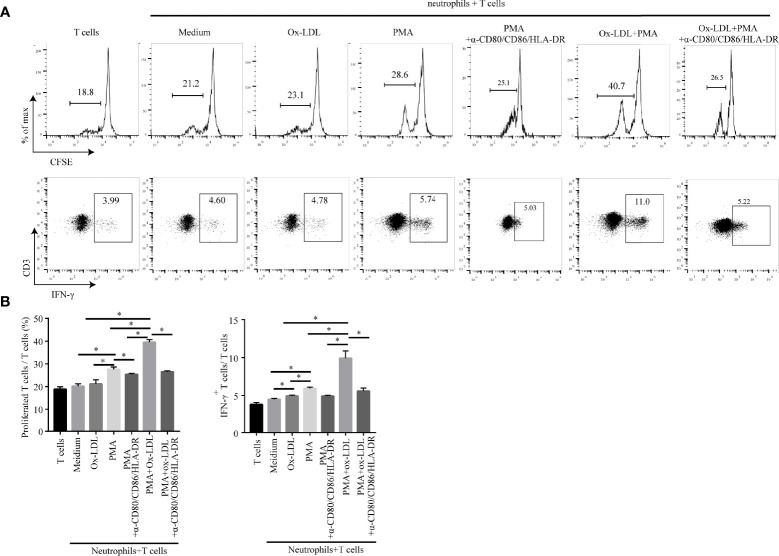
APC-like neutrophils increase T cell proliferation and IFN-γ production *in vitro*. Isolated CFSE-labelled peripheral CD3^+^ T cells of healthy donors were co-cultured for 5 days with neutrophils treated under different conditions: medium, ox-LDL, PMA, ox-LDL +PMA, with or without blocking antibodies (human HLA-DR/CD80/CD86 neutralization antibodies). Representative graphs **(A)** and statistical analysis **(B)** of T cell proliferation and IFN-γ production were shown (n=3). *p < 0.05.

APCs are the key players in the immune response since they are capable of presenting antigens to T cells thereby initiating T cell responses (38). To determine the role of APC-like neutrophils in T cell function, CFSE-labeled CD3^+^ T cells were co-cultured with conditioned neutrophils which were pretreated with ox-LDL plus PMA. Then, T cell proliferation and IFN-γ production were measured by flow cytometry. Neutrophil/T-cell co-cultures showed that APC-like neutrophils (induced by ox-LDL and PMA) significantly promoted the proliferation and IFN-γ production of T cells, which could be significantly attenuated by blockade of HLA-DR, CD80 and CD86 on neutrophils (**Figure 5**).

**Figure 6:**
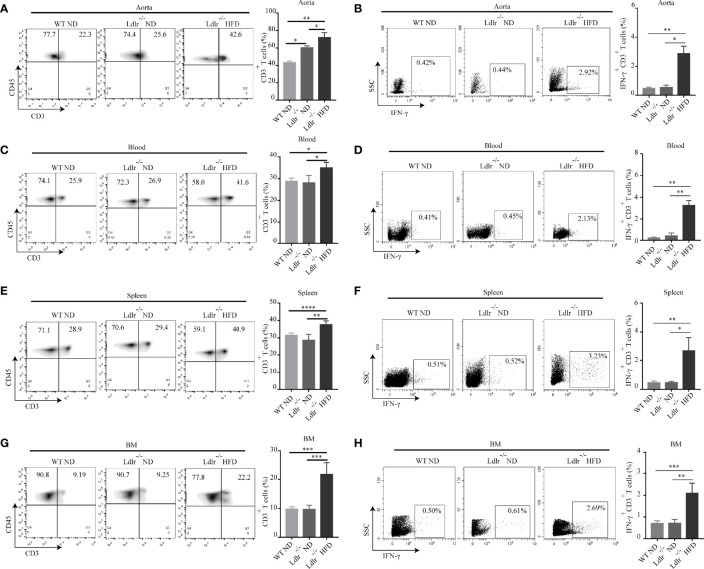
Percentages of CD3^+^ T cells and IFN-γ^+^ T cells are higher in LDLr^-/-^HFD mice compared to control groups. The distribution of CD3^+^ T and IFN-γ^+^ T cell in the **(A, B)** aorta, **(C, D)** blood, **(E, F)** spleen (SP), and **(G, H)** bone marrow (BM) of WT ND, LDLr^-/-^ND and LDLr^-/-^HFD mice were measured by flow cytometry. *p < 0.05, **p < 0.01, ***p < 0.001, ****p < 0.0001.

To verify these findings *in vivo*, we analyzed the distribution of T cells in atherosclerotic mice. Gate strategies of T cells and IFN-γ^+^CD3^+^ T cells were shown in **Supplementary Figure S6**. Consistently, we found that more CD3^+^ T cells were infiltrated in aorta of LDLr^-/-^ mice with HFD than control groups (**Figure 6A**). The percentage of IFN-γ^+^CD3^+^ T cells in the aorta of LDLr^-/-^ mice with HFD was also much higher than that of control groups (**Figure 6B**). Increased CD3^+^ T cells and IFN-γ^+^CD3^+^ T cells were found in blood, spleen and bone marrow of atherosclerotic mice as well (**Figures 6C–H**). The *in vitro* and *in vivo* results together suggest that APC-like neutrophils may play an essential role in T cell activation, but further research is needed to verify it.

### APC-Like Neutrophils Positively Correlate With T Cells in Hyperlipidemic Patients

**Figure 7:**
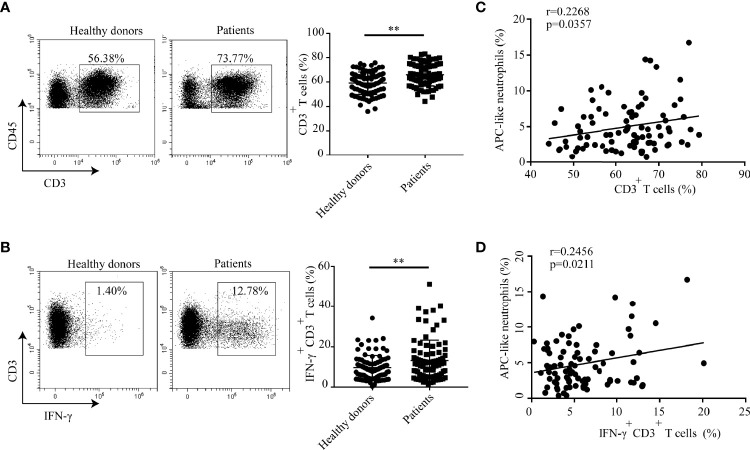
Percentages of CD3^+^ T cells and IFN-γ^+^CD3^+^ T cells are higher in hyperlipidemic patients than that of healthy donors. Percentage of **(A)**T cells and **(B)** IFN-γ^+^ T cell in blood of patients with hyperlipidemia and healthy donors were detected by flow cytometry. **(C)** Correlation analysis between APC-like neutrophils and CD3^+^ T cells in hyperlipidemic patients. **(D)** Correlation analysis between APC-like neutrophils and IFN-γ^+^ T cells in hyperlipidemic patients. In this set, HLA-DR^+^ neutrophils were defined as APC-like neutrophils. **p < 0.01.

The demographic and clinical characteristics of the patients with hyperlipidemia were outlined in **Supplementary Table S1**. The distribution of T cells in human peripheral blood was analyzed. The results showed that the percentage of CD3^+^ T cells in peripheral blood of hyperlipidemic patients was much higher than that in healthy donors (**Figure 7A**). Furthermore, a greater proportion of IFN-γ^+^ CD3^+^ T cells was found in blood of hyperlipidemic patients comparing to that of healthy donors (**Figure 7B**). Within the patient cohort, APC-like neutrophils were positively correlated with CD3^+^ T cells and IFN-γ^+^ CD3^+^ T cells, respectively (**Figures 7C, D**). The results were in accordance with that observed in neutrophil/T-cell co-cultures, which implies a stimulatory role of APC-like neutrophils in the early stage of human atherogenesis.

## Discussion

In this study, we have identified a unique neutrophil subset which expresses APC associated markers including HLA-DR (human)/MHC-II(mice), CD80 and CD86. We show that the percentage of APC-like neutrophils significantly increases both in hyperlipidemic patients and atherosclerotic mice. We uncover that ox-LDL play pivotal roles in the differentiation of PMA-activated neutrophils into APC-like phenotype. What’s more, APC-like neutrophils foster T cell response *via* HLA-DR, CD80 and CD86 *in vitro*, implying an immunostimulatory effect of APC-like neutrophils in atherosclerosis. Our clinical data also support the concept because there is a positive correlation between neutrophils and CD3^+^T cells and IFN-γ^+^ CD3^+^ T cells, separately, in hyperlipidemic patients. Despite the fact that neutrophils have previously been described in atherosclerosis, to our knowledge this is the first demonstration for the existence of APC-like neutrophils in atherosclerosis.

The involvement of neutrophils in the pathogenesis of atherosclerosis has recently received a lot of attention. However, most of the current studies are focused on the granule proteins and cytokines released by neutrophils in atherosclerosis (39). Here, we show that neutrophils in the blood of hyperlipidemic patients exhibited an APC-like phenotype characterized by expression of HLA-DR, CD80 and CD86. The same subset of neutrophils were also found in aorta, blood and spleen of atherosclerosis mice. This discovery is accordance with what found in inflammatory lesions (40) and allergic conditions (41), implying APC-like neutrophils may exist widely in inflammatory diseases including atherosclerosis.

In the progression of atherosclerosis, ox-LDL regulates the phenotype and function of various cells such as macrophages and T lymphocytes (42–45). However, there have been few studies focusing on the effect of ox-LDL on neutrophil phenotypes. Our work reveal that PMA-activated neutrophils can differentiate into APC-like neutrophils *via* ox-LDL.There are two possible reasons to explain this phenomenon. Firstly, because it is reported that ox-LDL induces NET formation in human neutrophils via toll like receptor (TLR)-PKC-IRAK-MAPK and NADPH-oxidase activation (46), we speculate that ox-LDL is likely to mediate the differentiation of neutrophils through TLRs; however, without PMA activation, the expression of TLRs on the surface of fresh neutrophils is extremely low, so ox-LDL alone cannot differentiate neutrophils into APC-like subset. The second explanation is that some inflammatory factors in atherosclerosis may work together with ox-LDL, playing a similar role to PMA *in vitro*, to regulate the differentiation of APC-like neutrophils. Further research is needed to validate these hypotheses.

The antigen presentation function of APCs has long been recognized as a pivotal component in T cell activation (40). HLA-DR/MHC-II-antigen crosstalk provides the first signal while costimulatory pathway provides the second signal of T cell activation (47). In this study, we show that APC-like neutrophils promote T cell proliferation and IFN-γ production through HLA-DR, CD80 and CD86 *in vitro*. In consistence with this, increased percentages of CD3^+^ T cells and IFN-γ^+^ CD3^+^ T cells are found in atherosclerotic mice and hyperlipidemic patients. Correlation analysis reveals a positive correlation between APC-like neutrophils and CD3^+^ T cells/IFN-γ^+^ CD3^+^ T cells. IFN-γ is the only member of type II IFN secreted mainly by T cells and macrophages (48). It is involved in the initiation and modulation of a variety of immune responses, many of which are pro-atherogenic (49). Atherogenic effects of IFN-γ have been shown in murine models where exogenous administration enhances atherosclerotic lesion formation while knockout of IFN-γ or its receptor reduces lesion size (50). Thus, APC-like neutrophils described here in atherosclerotic mice and hyperlipidemic patients may play a pro-atherogenic role through induction of IFN-γ by CD3^+^ T cells.

Atherosclerosis involves an ongoing inflammatory response. The atherosclerotic plaque consists of large amounts of inflammatory cells and the interaction of these immune cells plays a vital part in atherosclerosis progression. Our study identifies a novel pro-atherogenic APC-like neutrophil subset in atherosclerosis, and reveal that APC-like neutrophil subset contributes to T cell activation, which is consistent with our clinical correlation analysis. Thus, our study provides a new perspective for the immune theory of atherosclerosis and provides new targets for anti-atherosclerosis therapy.

## Data Availability Statement

The original contributions presented in the study are included in the article/**Supplementary Material**. Further inquiries can be directed to the corresponding authors.

## Ethics Statement

The studies involving human participants were reviewed and approved by Ethics Committee of Chongqing Medical University. The patients/participants provided their written informed consent to participate in this study. The animal study was reviewed and approved by the Animal Ethical and Experimental Committee of Chongqing Medical University.

## Author Contributions

TW, CY: conception and design, data analysis, manuscript revision. TZ: experiment conduction, data analysis, drafting the manuscript. QJ: data analysis, manuscript revision. WL: blood samples and clinical data collection. YW, YZ, XC: experiment conduction. TD, ZY, LM, and RY: technical support and editing. All authors contributed to the article and approved the submitted version.

## Funding

This work was supported by National Natural Science Foundation of China (81902922) and Chongqing Postdoctoral Science Foundation (cstc2019jcyj-bshX0076).

## Conflict of Interest

The authors declare that the research was conducted in the absence of any commercial or financial relationships that could be construed as a potential conflict of interest.

## Publisher’s Note

All claims expressed in this article are solely those of the authors and do not necessarily represent those of their affiliated organizations, or those of the publisher, the editors and the reviewers. Any product that may be evaluated in this article, or claim that may be made by its manufacturer, is not guaranteed or endorsed by the publisher.
