# Supplementary material for: Antigen-Presenting Cell-Like Neutrophils Foster T Cell Response in Hyperlipidemic Patients and Atherosclerotic Mice
Source: Front Immunol. 2022 Feb 17;13:851713. doi: 10.3389/fimmu.2022.851713 (PMC8891125; doi:10.3389/fimmu.2022.851713)
Supplement: Supplementary file 1 [file Presentation_1.pdf]

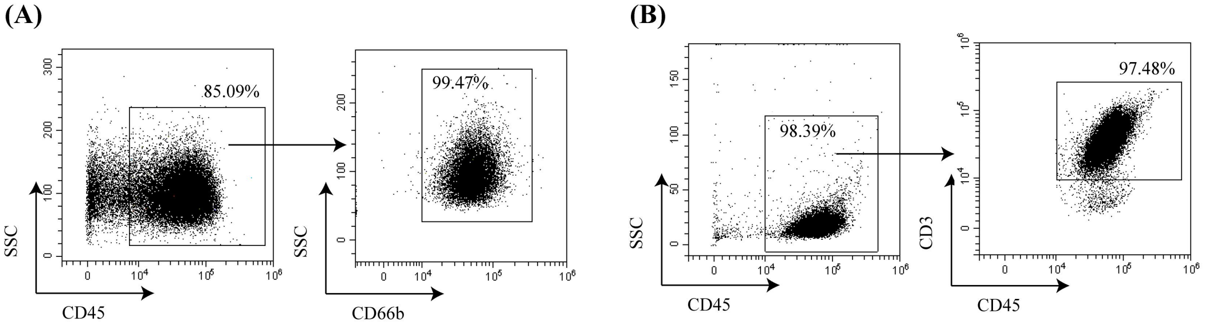

Supplementary Figure S1. The purity of neutrophils and T cells. (A) Purity of human CD66b$^+$ neutrophils isolated by density gradient centrifugation from peripheral blood of healthy volunteers. (B) Purity of T cells isolated by Easysep^{TM} human T cell isolated kit.

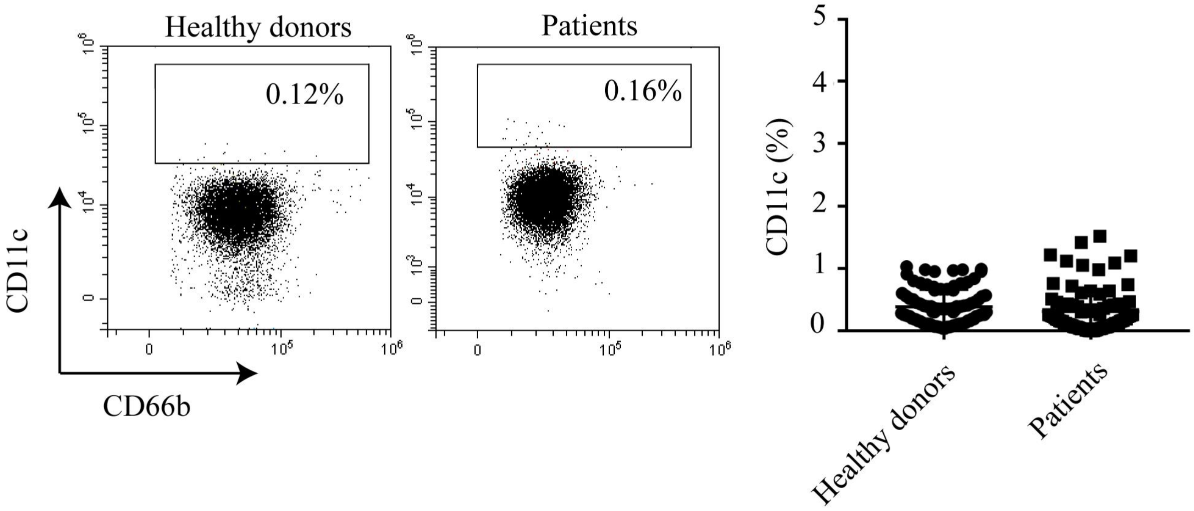

Supplementary Figure S2. Flow cytometry analysis of the percentages of CD11c^{+} neutrophils between patients with hyperlipidemia and healthy donors.

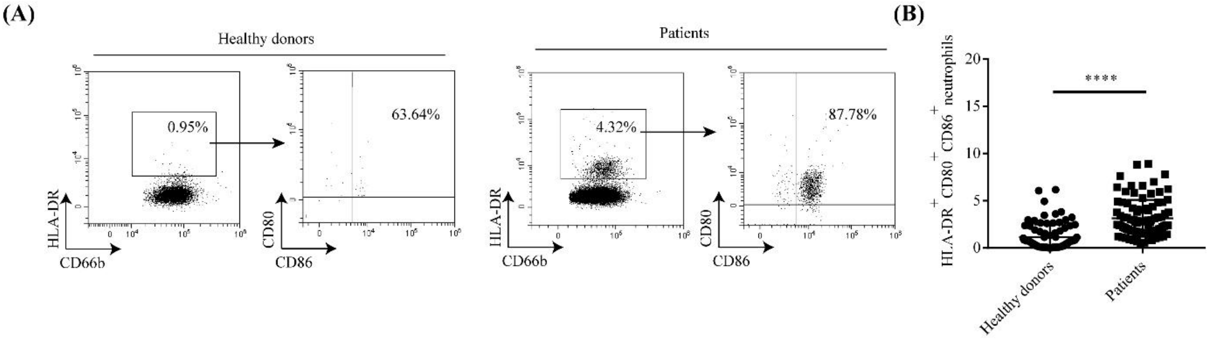

Supplementary Figure S3. Co-expression of HLA-DR, CD80 and CD86 on $CD66b^+$ neutrophils. (A) Gating strategies of $HLA\text{-}DR^+CD80^+CD86^+$ neutrophils in peripheral blood of hyperlipidemic patients and healthy donors. (B) Percentages of $HLA\text{-}DR^+CD80^+CD86^+$ neutrophils in $CD66b^+$ neutrophils. ****$p<0.0001$.

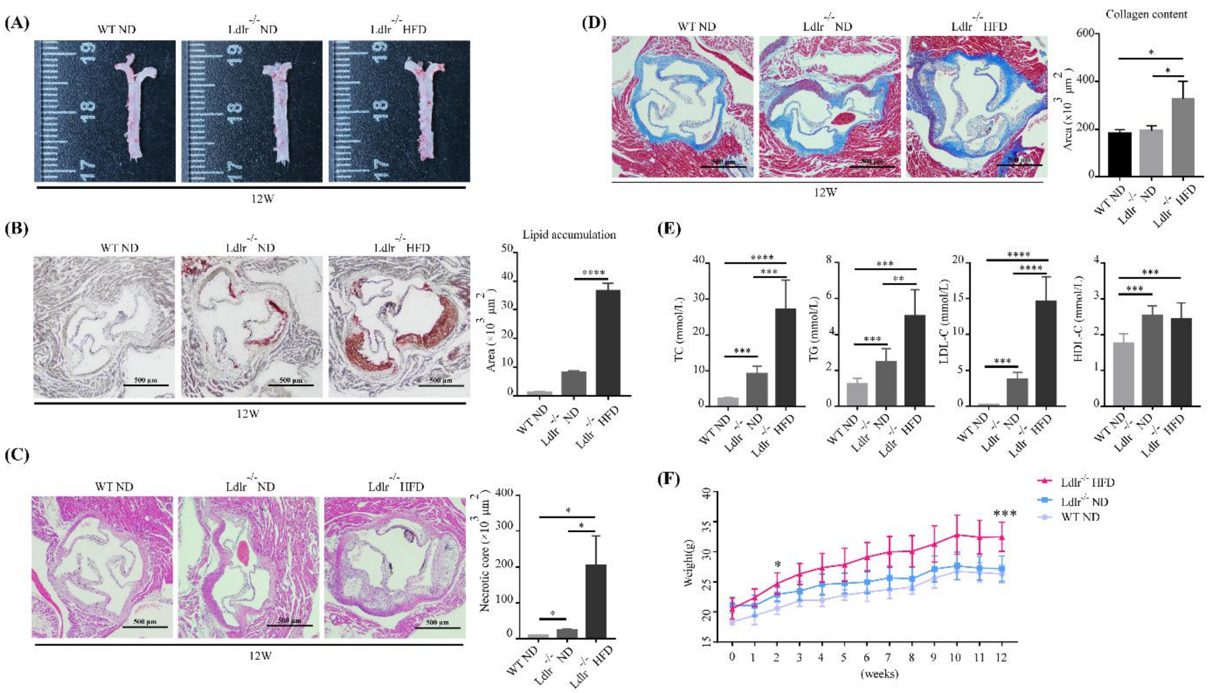

Supplementary Figure S4. High-fat diet feeding LDLr$^{-/-}$ mice induces atherosclerosis. (A) Representative enface photographs of aortas showing Oil Red O-stained atherosclerotic plaques. (B) Oil Red O staining and quantification of plaque area in aortic valves from WT ND, LDLr$^{-/-}$ ND, LDLr$^{-/-}$ HFD mice. (C) Representative histological analysis of aortic sinus from WT ND, LDLr$^{-/-}$ ND, LDLr$^{-/-}$ HFD mice stained with H&E and quantification of the necrotic area. (D) Masson trichrome staining and fibrosis quantification of aortic sinus from WT ND, LDLr$^{-/-}$ ND, LDLr$^{-/-}$ HFD mice. (E) Plasma TC, TG, LDL-C and HDL-C of WT ND, LDLr$^{-/-}$ ND, LDLr$^{-/-}$ HFD mice were measured. (F) Mouse weight curve of WT ND, LDLr$^{-/-}$ ND, LDLr$^{-/-}$HFD throughout the experiment. n=8. *$p<0.05$, **$p<0.01$, ***$p<0.001$, ****$p<0.0001$.

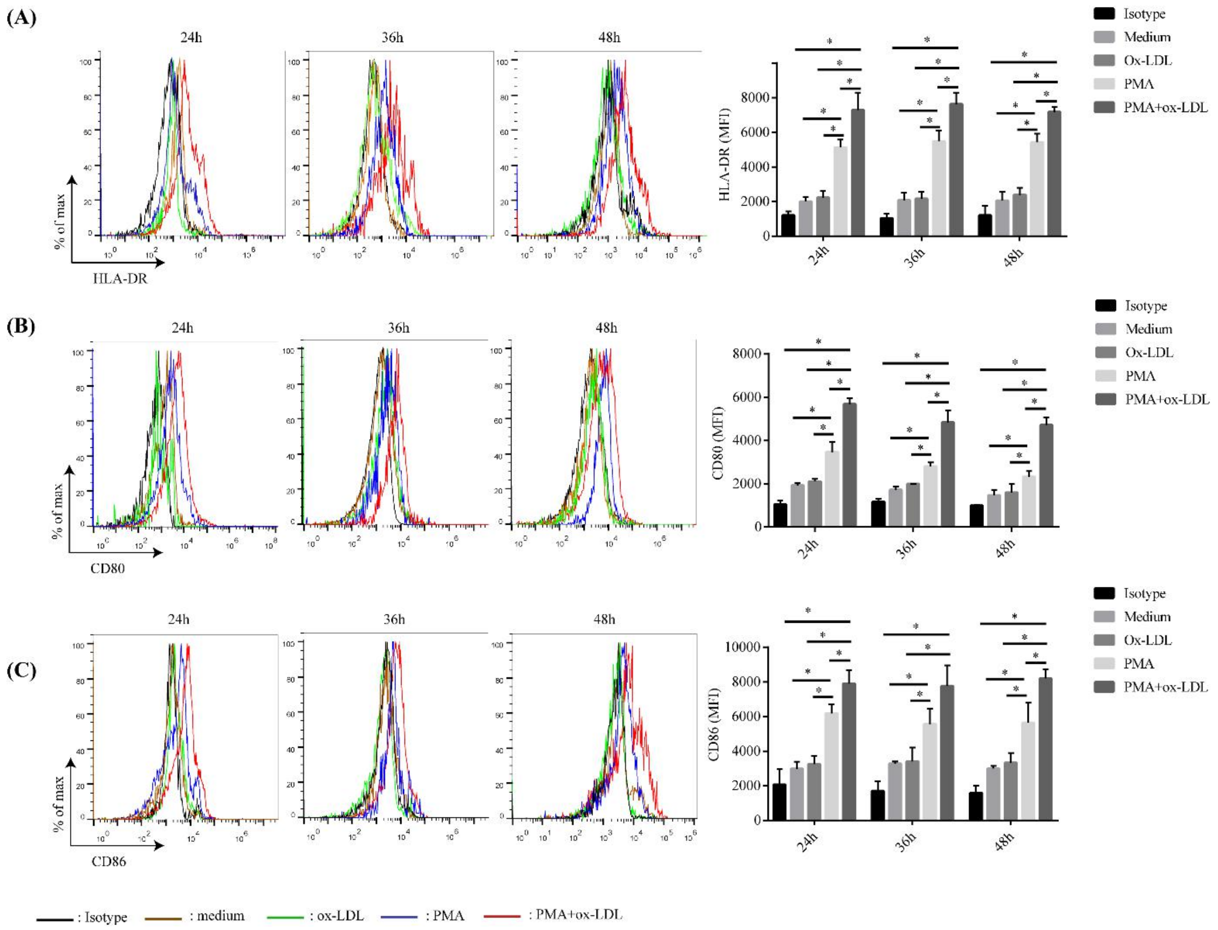

Supplementary Figure S5. Ox-LDL induces activated neutrophils to express HLA-DR, CD80, and CD86. Human-derived neutrophils undergo different treatment conditions: no treatment (brown line), 40 μg/ml ox-LDL alone (green line), 1 nmol/L PMA (blue line), 1 nmol/L PMA + 40 μg/ml ox-LDL (red line). After culturing for 24h, 36h, 48h, separately, expression level of (A) HLA-DR, (B) CD80 and (C) CD86 on neutrophils were analyzed by flow cytometry. Black line: isotype control. MFI: mean fluorescence intensity. Data presented in the bar graph are the mean ± SD of three experiments. *p<0.05.

(A)

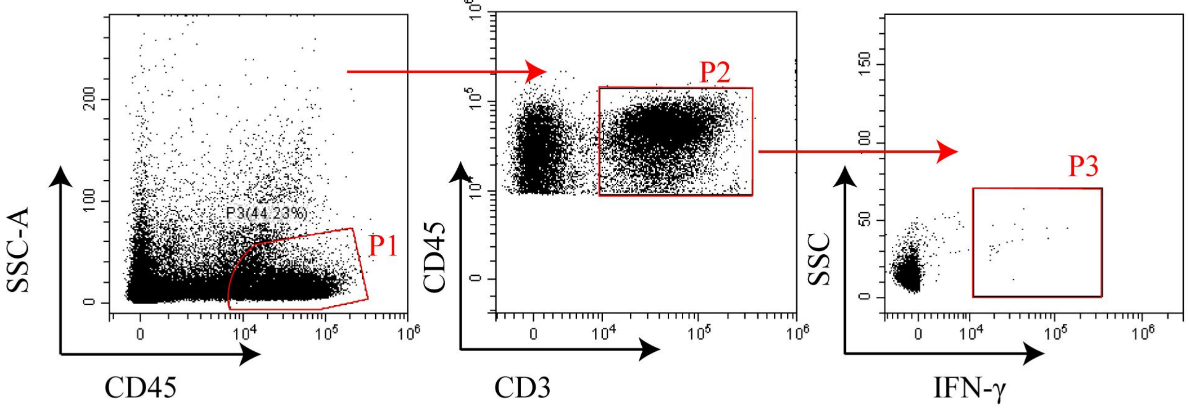

Supplementary Figure S6. Gating strategies of CD3+ T cells and IFN-γ+ CD3+ T cell subsets.

Supplementary Table S1. Clinical parameters of patients with hyperlipidemia

Variables	No. of patients
Gender (male/female)	41/49
Age (years; median, range)	50, 22-62
TC (mmol/L, ≤5.6/>5.6)	0/90
TG (mmol/L, ≤2.3/>2.3)	46/44
LDL-C (mmol/L, ≤3.37/>3.37)	12/78
HDL-C (mmol/L, ≤0.9/>0.9)	4/86
$HLA\text{-}DR^{+}$ neutrophils (%, median, range)	4.16, 0.39-19.42
$CD80^{+}$ neutrophils (%, median, range)	3.25, 0.35-13.94
$CD86^{+}$ neutrophils (%, median, range)	2.93, 0.41-24.40
$CD3^{+}$ T cells (%, median, range)	61.31, 12.06-83.06
$IFN\text{-}\gamma^{+}$ CD3 T cells (%, median, range)	11.93, 1.25-38.99
